# Archimedean Spirals Form at Low Flow Rates in Confined Chemical Gardens

**DOI:** 10.1021/acs.langmuir.2c00633

**Published:** 2022-05-20

**Authors:** Luis A. M. Rocha, Lewis Thorne, Jasper J. Wong, Julyan H. E. Cartwright, Silvana S. S. Cardoso

**Affiliations:** † Department of Chemical Engineering and Biotechnology, University of Cambridge, Cambridge CB2 3RA, U.K.; ‡ Instituto Andaluz de Ciencias de la Tierra, CSIC−Universidad de Granada, 18100 Armilla, Granada, Spain; ¶ Instituto Carlos I de Física Teórica y Computacional, Universidad de Granada, 18071 Granada, Spain

## Abstract

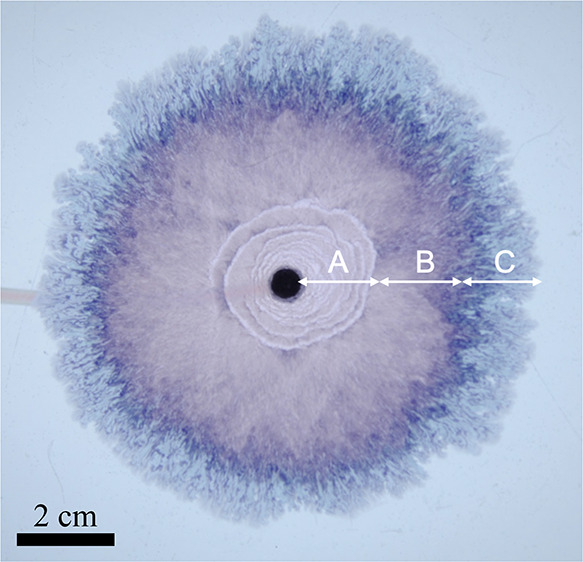

We describe and study the formation of confined chemical garden patterns. At low flow rates of injection of cobalt chloride solution into a Hele-Shaw cell filled with sodium silicate, the precipitate forms with a thin filament wrapping around an expanding “candy floss” structure. The result is the formation of an Archimedean spiral structure. We model the growth of the structure mathematically. We estimate the effective density of the precipitate and calculate the membrane permeability. We set the results within the context of recent experimental and modeling work on confined chemical garden filaments.

## 1 Introduction

Pattern formation is commonly observed in nature as a result of physical, chemical, or biological self-organizing processes.^1,2^ A remarkable example of such self-organizing patterns are chemical gardens, precipitate structures formed when a metal salt contacts with a solution of silicate, phosphate, carbonate, or many other anions.^3−5^ Various methods have been developed to grow these structures, which lead to a wide array of patterns and regimes;^6−14^ the one characteristic common to all is the formation of a semipermeable precipitate membrane separating two fluids which establishes a steep concentration and pH gradient. The earliest such experimental method is seed growth, which simply involves placing a solid crystal of a metal salt in a reservoir containing a silicate solution. The precipitation reaction forms a membrane surrounding the seed, across which a concentration gradient drives water into the seed through osmosis. Eventually, the increase in pressure causes the membrane to rupture, releasing a buoyant jet of the inner solution. This process then repeats, given the periodical rupturing and healing of the membrane by precipitation. The result is the formation of multiple vertical tubes; the resemblance of this pattern with the stalks of plants led to the name *chemical garden*.^3,15^ Novel methods were developed over time, allowing for better control of the experimental variables, and thus uncovering new chemical garden patterns. If a metal salt solution is pumped into the reservoir rather than introduced with a seed, vertical tubular structures are still formed, which can range from thin jet-like filaments, to wider oscillating tubes, to even wider bulbous structures.^16,17^ These different regimes were found to be due to the density difference between the lighter injected fluid and the denser host solution.^16,17^ Further control over the formation of these precipitate patterns is achieved with Hele-Shaw cells:^18−22^ quasi two-dimensional micro reactors where one of the reactants is injected into the other. This method reveals a truly remarkable array of different patterns, shaped by the viscosity differences of the fluids as well as the local velocity. Such regimes include “spirals”, “flowers”, “worms”, and “filaments”.^23,24^ These filaments are one of the most noteworthy regimes and occur when the concentrations of the reactants are both high and the injection flow rate is above a certain threshold. They consist of thin tubular structures, with an active tip that periodically changes direction after short rectilinear paths, resulting in a zigzag pattern.^25−29^ The motion of these patterns has been modeled according to the oscillatory dynamics of the membrane at the tip of the filament.^27^ Below the flow rate threshold, no filaments are observed and the precipitate spreads radially from the nozzle.^25,27,30^ In this work, Hele-Shaw cells are used to grow chemical gardens with very low flow rates but high concentrations of the reactants, sodium silicate, and cobalt chloride. The evolution of the radial structures formed is modeled, and a novel pattern is observed, an *Archimedean Spiral*;^31^ this appears to be the result of the simultaneous growth of two distinct regimes.

## 2 Experimental Methods

**Figure 1:**
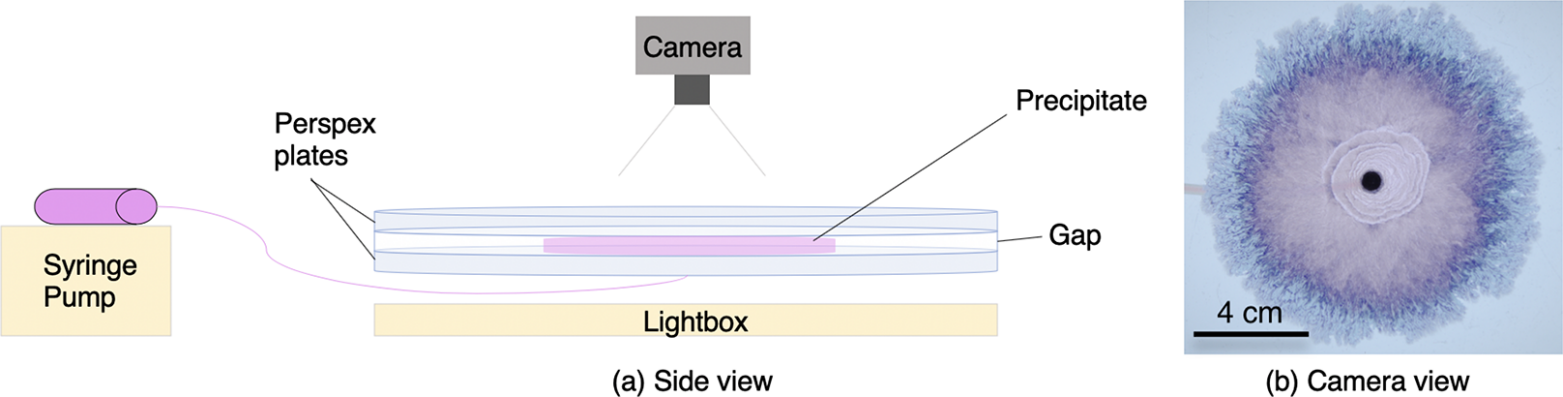
Schematic of the experimental setup.

A horizontal Hele-Shaw cell was used for experiments (Figure 1). The setup consisted of two circular perspex plates (30 cm in diameter) separated by rubber spaces 0.5 mm thick and placed horizontally over a light pad for illumination. The cell was initially filled from a center injection nozzle with a host solution of sodium silicate (3.13 M), prepared by dilution in water of a commercial solution. A displacing solution of cobalt chloride (0.63 M) was then injected into the cell. This solution was prepared by dissolution of the powder in water. A syringe pump was used to pump both solutions into the cell. Photographs were taken from above during each experiment at 5 s intervals with a Nikon D300s digital single-lens reflex camera (DSLR, 4288 × 2848 pixels) with a Hoya circular polarizing lens filter. The images were analyzed with MATLAB to determine the growth of the precipitate area with time. This involved binarizing the image to black and white, calculating the area of precipitate in pixels, and then converting to cm^2^.

## 3 Experimental Observations

### 3.1 Regime Characterization

**Figure 2:**
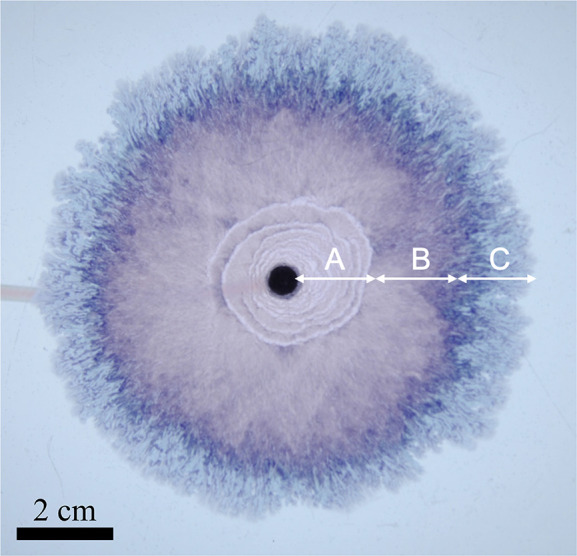
Different regimes observed during an experiment. The structure spreads radially from the nozzle at the center; different patterns emerge as the local velocity decreases: (A) Archimedean spirals/candy floss, (B) candy floss, and (C) lichen/worms.

As the cobalt chloride solution is injected into the host solution of sodium silicate, precipitate patterns grow radially from the center injection point. As the structure grows, various different patterns can be observed. The different regimes observed are presented in Figure 2.

A new regime was observed, *Archimedean spirals*, together with another possibly novel morphology, termed here *candy floss*. Candy floss growth is characterized by a uniform and radial seeping of precipitate, with a fluffy pink color, hence the name. This pattern is similar to the moss regime already reported in the literature,^30^ which is also the first regime to appear in similar experiments, but with a more homogeneous pink color. The moss pattern is described as having the features of compact fibers, with large inner fingers and a violet-blue color,^30^ quite different from the candy floss morphology. These differences may be due to the different concentrations of the reactants in the two cases, as well as the slightly lower flow rate used to generate the moss pattern (∼1 μL s^–1^). It is possible that moss and candy floss are mere variations of the same regime; however, given the differences, the pattern observed in this work is referred to as *candy floss* in this paper.

Archimedean spirals were found to grow together with candy floss, for all injection flow rates of 3.3 μL s^–1^ (0.2 mL min^–1^) and above. These spirals consist of a clear line of precipitate growing around the edge of the existing candy floss structure. Because these two patterns cogrow, the lines growing around the edge are approximately equally spaced on successive turnings, thus forming an Archimedean spiral. Indeed, such a spiral is defined as a curve increasing its distance from the origin at a constant rate, along a line that rotates at a constant angular velocity (expressed mathematically as *r* = *bθ* with *b* as the filament width); that is similar to the process observed experimentally in the chemical garden. (The angular velocity of the precipitate spiral is not constant, but the velocity is. The result is ultimately the same, the formation of the chemical garden just slows down with time.)

The equation for the arclength of an Archimedean spiral is
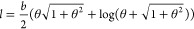
1If we assume the spiral grows at a constant speed and take the derivative with respect to *t*, we obtain
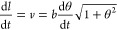
2This first-order nonlinear ordinary differential equation may be integrated to derive an implicit expression relating θ and *t*:
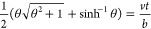
3defining θ = 0 at *t* = 0. A similar implicit expression for *r* and *t* may easily be obtained from the equation for an Archimedean spiral:
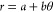
4

Here, we consider the curve produced by a physical process in which material is injected into a 2D setup, so having negligible width. At the beginning of the process, a circle of a certain diameter forms. At this point the circle becomes unstable and material starts to leak out to form a filament that wraps around itself. The process then continues as an advancing filament of fixed width of this material hugs itself. Before the amount of material injected is sufficient to form a filament, precisely what form this curve takes depends on how we might describe in detail the initial injection. However, once past this initial stage, a self-hugging filament of fixed width is described by a spiral; the distance between whose turns is constant. Such a curve is an Archimedean spiral,^31^ or its close relative, the involute of the circle.^32,33^ These two curves are practically indistinguishable except very close to the origin (the Archimedean spiral is in fact the pedal curve of the involute of a circle, with the center as pedal point^34^). Therefore, we refer for simplicity to the curve as an Archimedean spiral. The way Archimedes formed his spiral is to think of a point moving at a constant rate along a straight line that rotates around a point lying on that straight line. Imagine something like the path an ant makes when it walks along the hand of a clock from the center outward. If one thinks of the spiral being constructed thus, if you consider the ant’s speed relative to the fixed clock face, it increases as it moves outward. But you obtain the same spiral, only being drawn at the constant speed, rather than a constant angular velocity, if a second ant marches round and round the clock face from the center at a constant speed spiralling outward while following the trajectory left by the first. This is our case. In fact, Archimedes himself is interested in his original exploration of the spiral^31^ in the areas between successive turns, which he found to be in arithmetic progression,^35^ and these areas tell us how long it takes for our filament to complete each succeeding coil.

**Figure 3:**
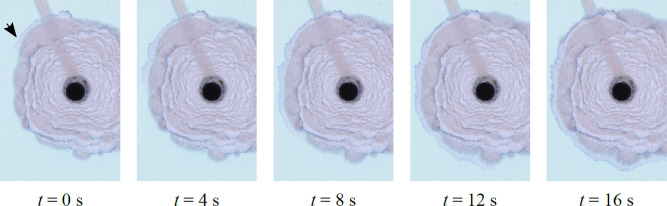
Time sequence of photographs showing the motion of a spiral segment. The arrow points to the starting point of the line; it then grows counterclockwise. The segment forms at the edge of the candy floss region, which separates the various curves of the spiral. The Archimedean spiral and the candy floss grow simultaneously; candy floss grows in all directions (note how it eventually even starts leaking from the outside of the spiral itself, starting at *t* = 12 s), and the spiral evolves independently, adding new sections to its moving tip in a linear fashion.

The expanding candy floss does not spread as a perfect circle, however, so the path of the precipitate spiral may slightly deviate from a perfect Archimedean spiral (Figure 3). In a normal Archimedean spiral, the radius increases in so-called arithmetic progression, by a constant increment for each turn; here that is so, but with a deal of statistical noise. Nevertheless, we consider this term effectively communicates the mechanism of growth, while distinguishing it from other confined chemical garden spirals.^18^

**Figure 4:**
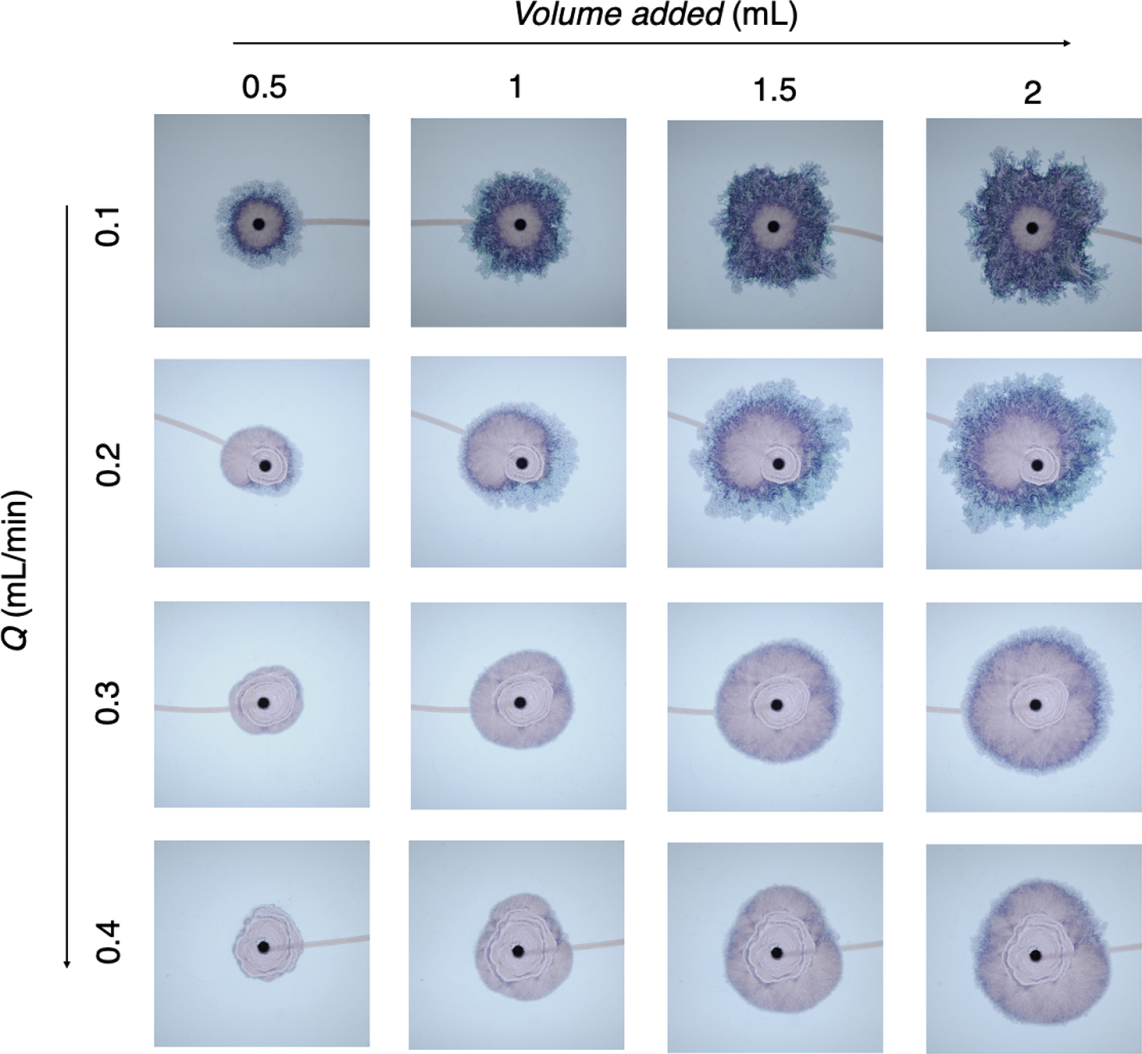
Sequence of photographs of precipitate patterns formed under different injection flow rates (1.71–6.7 μL s^–1^; 0.1–0.4 mL min^–1^) for different volumes of injected cobalt chloride (field of view: 9.7 × 9.7 cm).

Candy floss growth eventually dominates over the spirals, these cover a larger area with increasing *Q*. For *Q* ≤ 1.7 μL s^–1^, no spirals are observed and only candy floss is present, which generally grows symmetrically. The spirals can lead to an asymmetric growth of the precipitate in later stages, as shown for *Q* = 3.3 μs ^–1^ in Figure 4. After the candy floss regime, the precipitate growth transitioned to patterns of lichen and worms, as already described in the literature. These are characterized by a blue-green color and a wavier perimeter. The transition to new regimes always occurred at a larger radius of precipitate for higher injection flow rates.

### 3.2 Effect of Local Velocity

**Figure 5:**
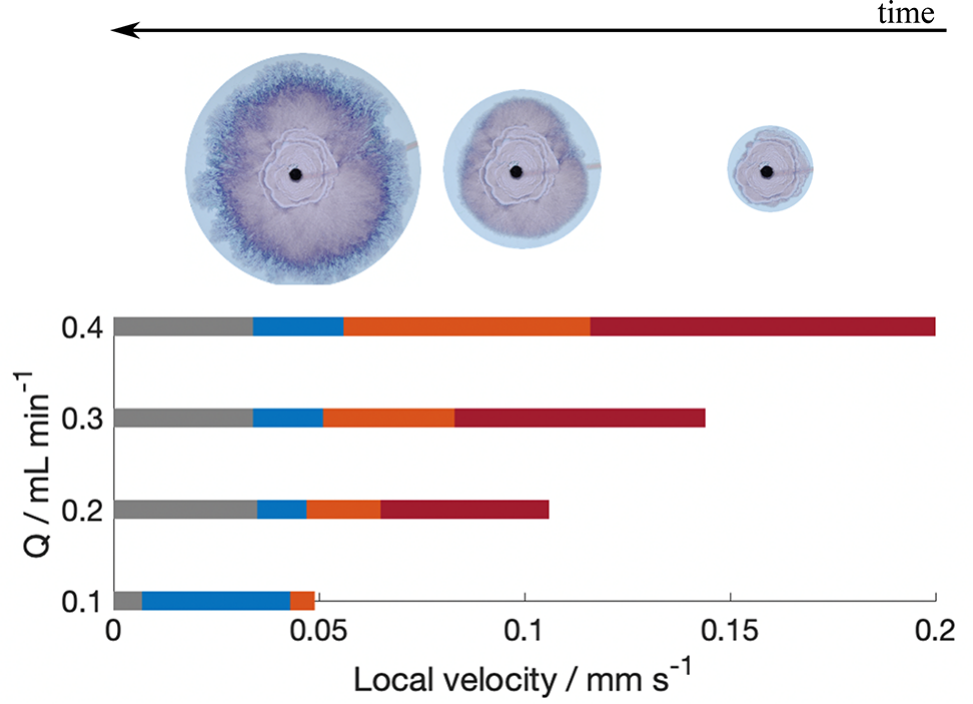
Pattern regime formed at calculated local velocities for different flow rates. Red bars, Archimedean spirals; orange bars, Candy-floss; and blue bars, lichen/worms regimes. Images above bars also indicate regime pattern. Transition to gray bars signify the end of the experiment.

The appearance of the different growth regimes is dependent on the local velocity at a given time.^30^ Because the flow rate of cobalt chloride injected into the cell is constant, the local velocity *u* = *Q*/(2*πLr*) decreases with radius *r* from the center injection point as the structure grows. Figure 5 shows the different patterns observed as a function of local velocity, for each *Q* tested. As the structure grows and the local velocity at its periphery decreases, higher flow rate patterns become unstable and new regimes emerge: filaments turn into Archimedean spirals, spirals stop and candy floss dominates, and eventually candy floss turns into lichen/worms.

**Figure 6:**
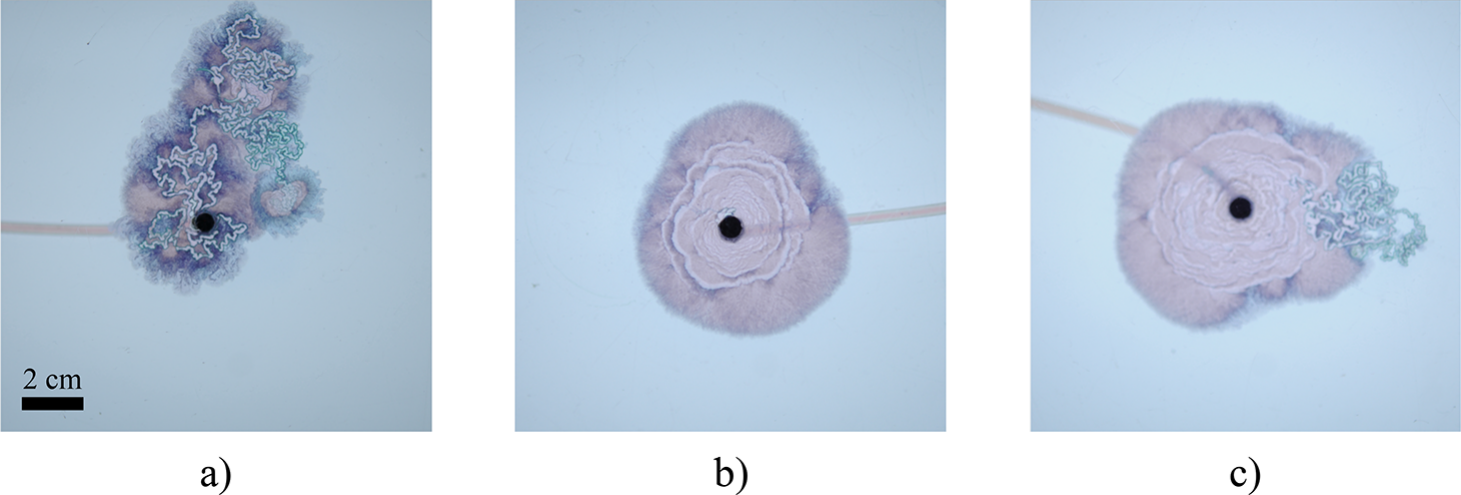
Photographs of precipitate after 175 s for three repeat experiments at *Q* = 6.7 μL s^–1^(0.4 mL min^–1^): (a) leaking from filaments; (b) spirals, no filaments; (c) filaments from spirals. In spite of the same experimental conditions, different results may be obtained. This is commonly observed in chemical garden experiments in Hele-Shaw cells; there exists a transition region between regimes where any one pattern may be observed. The fact that filaments are formed under the same conditions as spirals, or even emerging directly from them, suggests spiral segments may correspond to a filament wrapping around the candy floss structure; this would make them very similar to when filaments start leaking inner fluid from the walls (as shown in panel a). Spirals would then correspond to filaments self-hugging the leaking inner fluid.

Multiple regimes may be observed simultaneously, with the secondary patterns appearing in cogrowth always corresponding to lower local velocity regimes.^27,30^ It is common, for instance, for filaments to start leaking from the walls^27^ and for Archimedean spirals to appear together with candy floss. This implies that the local velocity may not be constant all across the perimeter of the precipitate, and it may decrease locally because of physical barriers caused by asymmetries in the precipitate morphology.^30^ The most noteworthy cases of cogrowth involve filaments, which require the highest local velocity. As initially expected, one can observe that filaments leak from the walls and then transition to candy floss, as shown in Figure 5. However, it is also possible for spirals to lead to filaments, as presented in Figure 6. This suggests that spiral growth may be very similar to filaments because the latter can emerge seamlessly from a loop of a spiral.

In addition, these data show that while it is often possible to predict which regimes will appear for a particular injection flow rate, there are still rather wide transition regions between regimes, where any of these patterns may emerge.^27^

**Figure 7:**
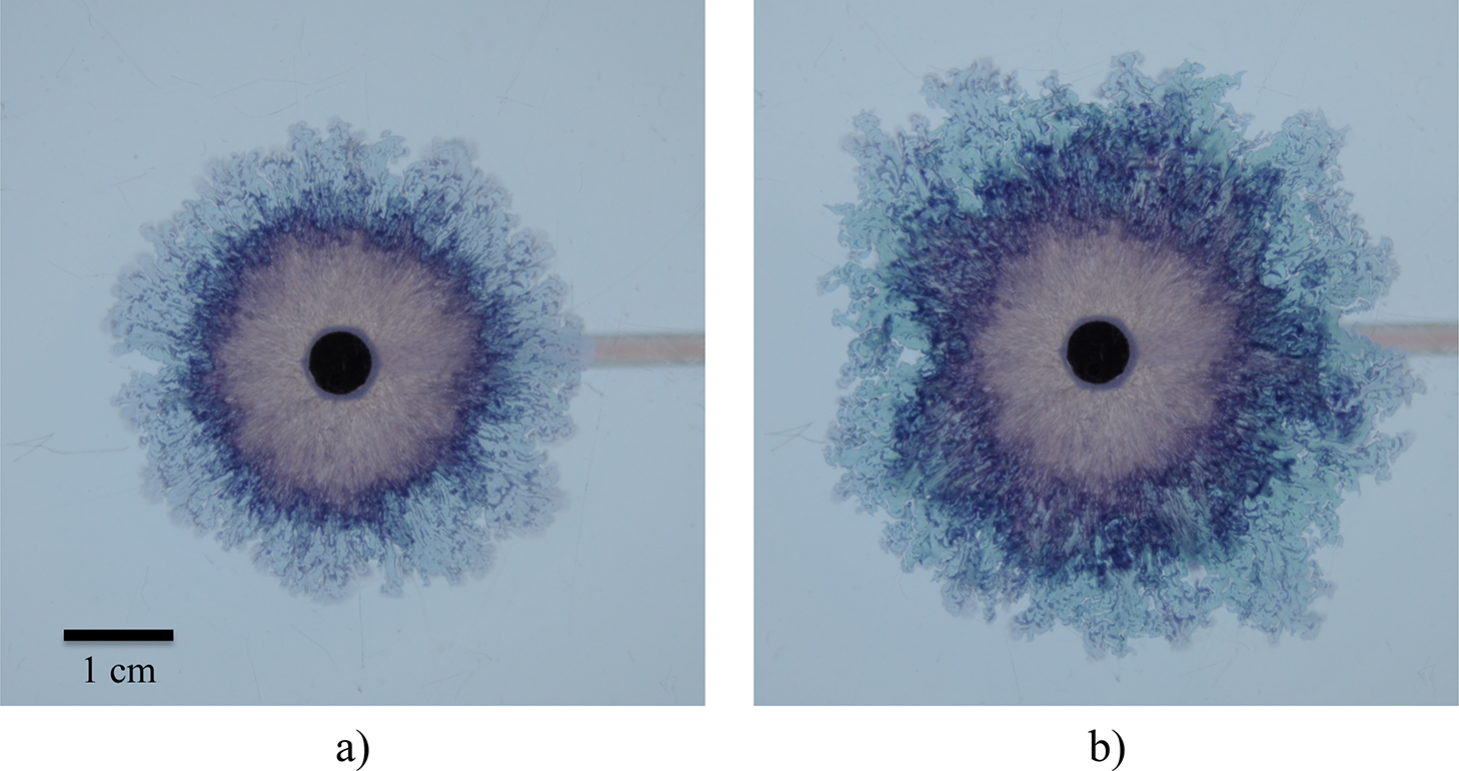
Thin dark annulus of secondary precipitate is apparent in the worms/lichen growth phase (see panel a). At a later time (b), the dark annulus of secondary precipitate has expanded through internal reaction zones.

**Figure 8:**
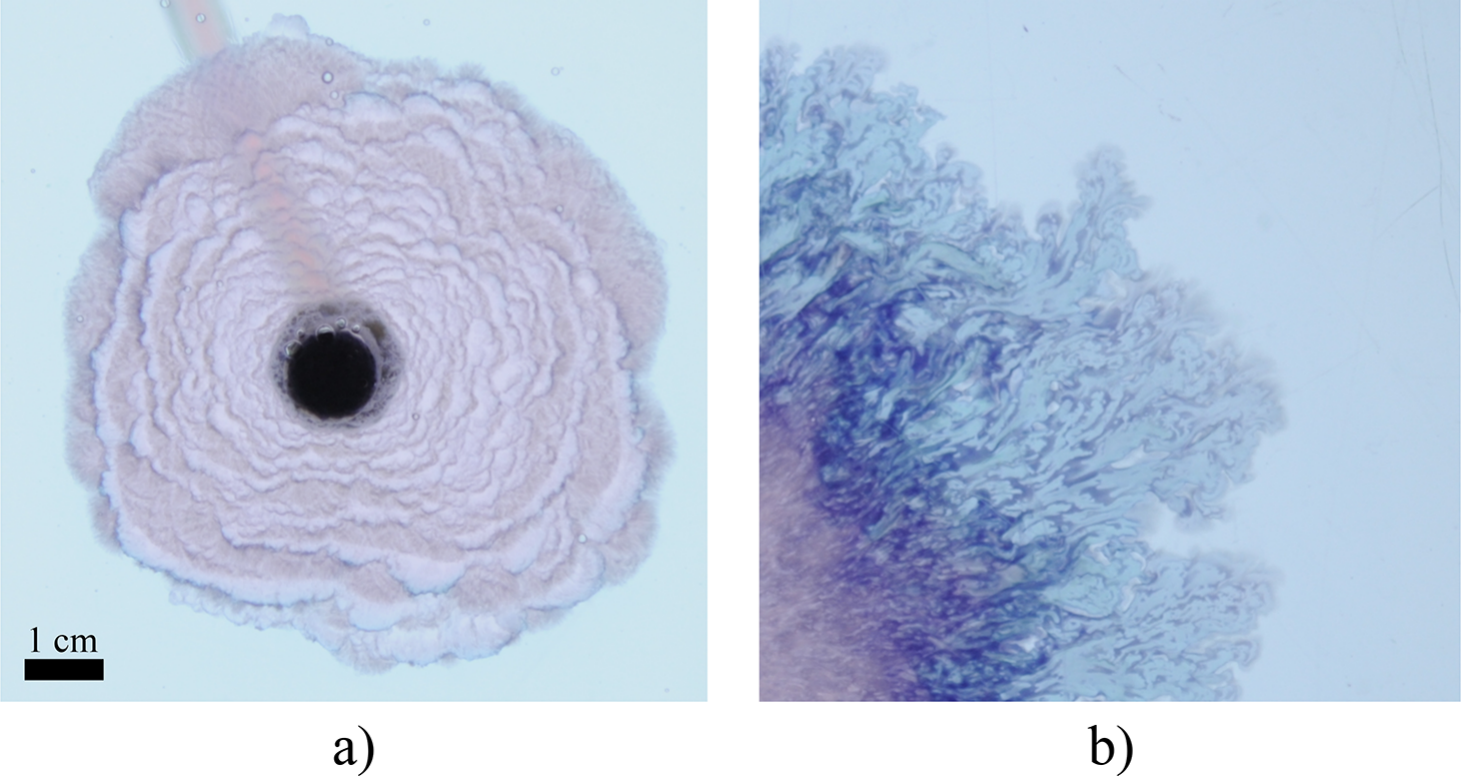
(a) Close-up of precipitate growth with the candy floss and Archimedean spiral regimes. The spiral segments sometimes are broken up into shorter ones by the expanding candy floss. These segments exhibit some bumps along their outline, possibly an indication of oscillatory dynamics similar to chemical garden filaments. (b) Detail of the lichen regime, which does not grow in a uniform manner. As a result, inclusions of outer solution may form within the precipitate, reacting with the inner solution at a later stage of the experiment. These areas are difficult to detect rigorously with image analysis, introducing uncertainties in the estimation of the effective density.

Cogrowth can also appear as internal reaction zones in the precipitate structure. During the lichen/worms phase, a darker blue layer within the main front can be seen to expand at the same time as the whole precipitate area grows, as shown in Figure 7. One possible explanation for this are the inclusions of unreacted sodium silicate left behind during the worms phase, presented in Figure 8b. These react at later times as more cobalt chloride flows through the system and is fed to the external surface of the precipitate. This effect complicates the modeling of the lichen/worms phase because the quantity of cobalt chloride that reaches the outer edge of solid and that contributes to the expansion of the structure is reduced. It also adds an error into the estimation of the precipitate area through the binary image processing method because these pockets of sodium silicate are difficult to detect automatically.

### 3.3 Effective Density Estimation

One important parameter when studying the growth of these chemical garden regimes is the density of the precipitate. Given how the precipitate membrane formed in the reaction is a hydrous, porous material, an experiment was conducted to estimate the effective density of the chemical garden structure. The experiment involved injecting known quantities of both solutions into the cell, first the silicate one and then cobalt, with known densities ρ_Si_ and ρ_Co_. With image analysis it is possible to determine the volume of sodium silicate before and after reaction, as well as the volume of precipitate formed. Assuming negligible density changes in the liquids and that the osmotic flow of water out of the chemical garden did not dilute the sodium silicate solution, the effective density can be calculated as
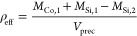
5Here, the subscript 1 refers to the mass of the reactant before the injection of cobalt chloride (and thus before reaction has taken place) and the subscript 2 refers to the end of the experiment, after the reaction is complete. Thus, *M*_Si,1_ is the mass of silicate injected into the cell at the start, *M*_Co,1_ the mass of cobalt injected after, and *M*_Si,1_ the mass of silicate not consumed by reaction and advected by the cobalt solution. *V*_prec_ is the volume of precipitate, measured with image analysis and assumed to fill the entire gap of the cell. The density was estimated to be ρ_eff_ = (1.19 ± 0.07) × 10^3^ kg m^–3^, for a precipitate grown at injection rate *Q* = 0.3 mL min^–1^. The error in the measurement is due to any solid formed within the nozzle being unaccounted for, as well as uncertainty regarding the moment when the solid started spreading in the cell. The effective density is much closer to the density of water than that of cobalt silicate crystals, suggesting a highly porous structure. Assuming that the only species present are the 0.63 M cobalt chloride solution and the crystals, which have densities of ρ_Co_ = 1070 kg m^–3^ and ρ_crystals_ = 4600 kg m^–3^, respectively, then ρ_eff_ = ρ_Co_(1 – ε) + ρ_crystals_ε, where ε is the liquid volume fraction. Solving this equation leads to ε = 0.97 ± 0.02.

Densities of various dry cobalt-silicate chemical garden pellets have been reported to lie in the range of 240–580 kg m^–3^,^22^ which correspond to porosities of 0.95–0.87, assuming the same density for the cobalt silicate crystals. The porosity estimated here is thus of a similar magnitude as these previous findings. The fact that the pellets have a lower porosity is also to be expected because these were grown over a much longer time scale of 1–2 h, instead of the few minutes for the experiments described in this study. In addition, these pellets were grown without injection, giving the solid more time to grow denser and without being spread by injection.

## 4 Mathematical Modeling

### 4.1 Precipitate Growth

**Figure 9:**
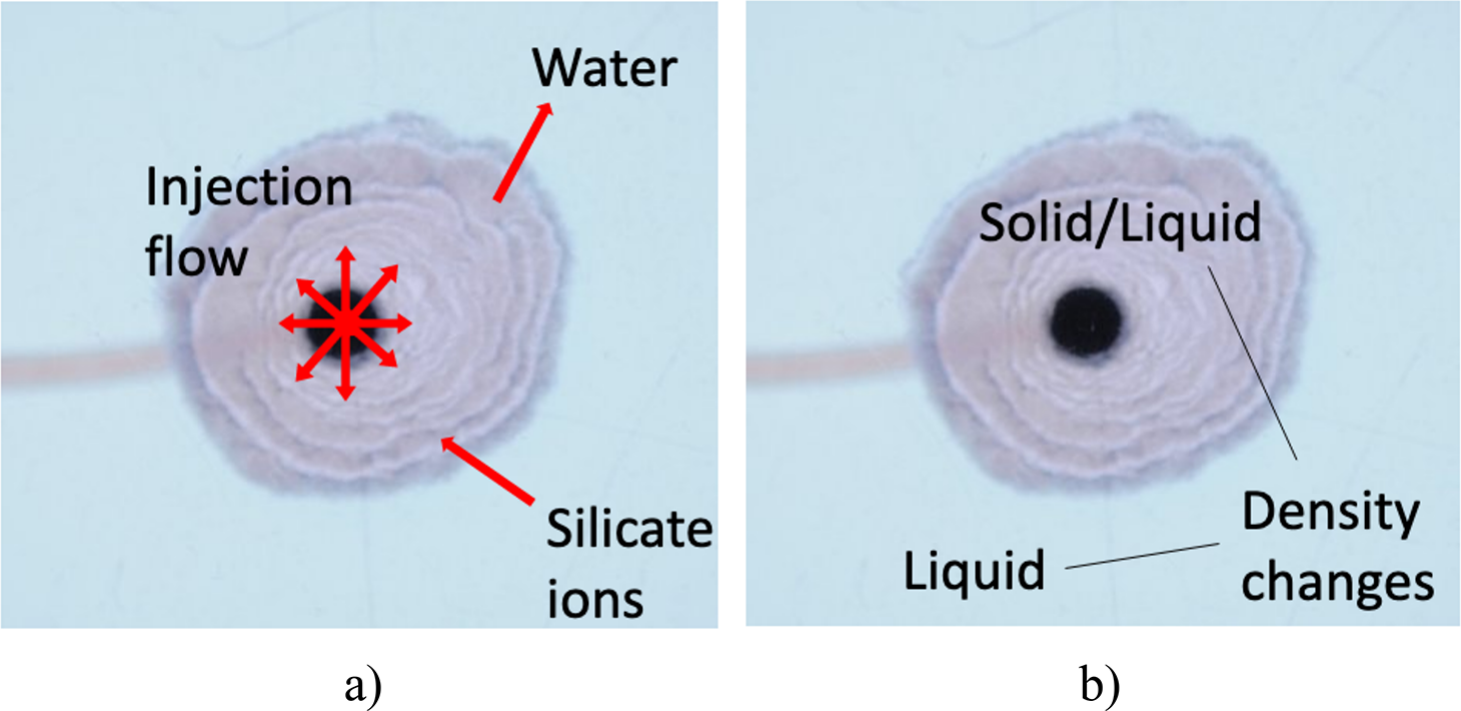
(a) Schematic of the mass flows into and out of the precipitate structure. Injection feeds cobalt chloride through the nozzle at the center; silicate ions are driven from the outer fluid and consumed in the precipitation reaction to form a membrane; the concentration gradient established across the membrane drives water into the structure through osmosis. (b) Density changes occur in the system: the reaction forms a precipitate product with a higher density than the liquid reactants, and the overall structure thus consists of the injected inner liquid and the precipitate product, with an effective density ρ_eff_. The consumption of silicate ions in the reaction also leads to a decrease in the density of the outer fluid, which is assumed to be negligible here.

By applying conservation of mass to this system, it is possible to model the growth of the precipitate structure during an experiment. This involves taking into account the effects of injection, chemical reaction, osmosis, and density change. The in/out flows and phase changes are illustrated in Figure 9.

These different contributions can be expressed mathematically as follows: 1. **Injection:** a cobalt chloride solution is continually injected into the cell with a syringe pump. The mass flow rate is thus
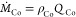
6

2. **Osmosis:** the precipitation reaction forms a semipermeable membrane, which allows the passage of water molecules but inhibits the flow of metal ions. Given that a 0.63 M solution of cobalt chloride is injected into a more concentrated 3.13 M sodium silicate host solution, an osmotic pressure is created that drives water out of the chemical garden. The velocity of water  can be approximated with Darcy’s law as follows:
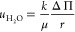
7where *k* is the precipitate membrane system permeability, μ the viscosity of water, *r* the average radius of the precipitate system, and *ΔΠ* the osmotic pressure. The osmotic pressure can be estimated from the following equation:
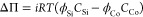
8where *i* is the dimensionless van’t Hoff index, *R* the molar ideal gas constant, and *T* the temperature in Kelvin; ϕ_Co_ and ϕ_Si_ are the activity coefficients of CoCl_2_ and Na_2_SiO_3_, respectively, and *C*_Co_ and *C*_Si_ are the molar concentrations of CoCl_2_ and Na_2_SiO_3_, respectively. The values for *i* and ϕ_*j*_ may be found in the literature;^36,37^ here, *i* = 1, ϕ_Si_ = 0.3, and ϕ_Co_ = 0.47. The permeability *k* of the precipitate membrane was initially unknown, and as a result was a fitting parameter in the model. Permeability is expected to vary with time as the membrane grows thicker;^38,39^ in this case, it should assume a value higher than that published for pellet growth (longer time scale for membrane formation) but lower than the permeability of a membrane at the tip of a moving filament (shorter time scale for membrane formation). The mass flow of water out of the system is thus
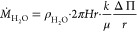
9where *H* is the gap between the plates of the Hele-Shaw cell.

3. **Reaction:** the two solutions react to form a hydrous precipitate of cobalt silicate
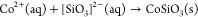
a

The reaction thus leads to the incorporation of silicate ions into the precipitate system, which will be limited by the number of cobalt ions, because sodium silicate is in excess. Assuming an instantaneous, complete, and irreversible reaction, the mass flow of silicate ions into the solid is
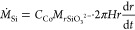
10Combining all these contributions, we obtain the following differential equation for the rate of change of mass of the solid:
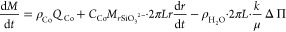
11where ρ_Co_ is the density of cobalt chloride solution, *Q*_Co_ the injection flow rate of cobalt chloride solution,  the molar mass of SiO_3_^2–^ ions, and  is the density of water. The solid structure formed during each experiment is composed of the precipitate cobalt silicate membrane as well as a liquid component flowing through it. The whole system is thus assigned an effective density ρ_eff_, and the total rate of change of mass is
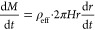
12If we combine eqs 11 and 12, we obtain
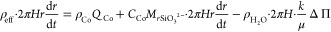
13which can be solved to give
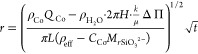
14This gives the radius of the precipitate structure as a function of time, considering the contributions of injection, osmosis, reaction, and density change. If reaction and osmosis are neglected, then ρ_eff_ ≈ ρ_Co_, reducing eq 13 to
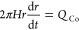
15which would simplify the temporal evolution of *r* to
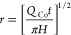
16This equation describes the evolution of the system considering only volume conservation, where the injected solution fills the cell gap entirely, as a cylinder of radius *r* and height *H*. Reaction and osmosis are thus corrections to this expression.

### 4.2 Archimedean Spiral Dynamics

The Archimedean spiral pattern is a novel chemical garden regime observed in this work. The thin spiral segments appear to grow from their tip, by rotating around the expanding candy floss structure. Filaments were observed to grow directly out of the spiralling segments; these exhibit the same color and width as the filaments, as shown in Figure 6. The spirals evolve together with the candy floss growth; this process is reminiscent of filaments leaking inner fluid.^27^ Indeed, once filaments start leaking from their side walls the flow rate reaching the tip decreases, and eventually their growth stops (a minimum flow rate is required to form a filament, which varies slightly with the chemical system^27^). A similar behavior is found in the spirals: their growth eventually stops as the leaking/candy floss dominates, and no spirals are even observed at lower flow rates. Additionally, it often happens that the final segment of the spiral is further away from the other curves of the spiral than expected, such as the example of Figure 2. This indicates that the spiral tip is advancing more slowly and is thus pushed away further as the candy floss area inflates. This behavior would be analogous to regular filaments slowing down and eventually stopping as all the inner fluid escapes through leaking. Such observations strongly suggest that the spirals consist of filaments wrapping around spreading precipitate through a self-hugging mechanism. Indeed, chemical garden filaments have been observed to follow the path of a pre-existing one when approaching and colliding with it. Therefore, it is possible that the dynamics of these chemical garden Archimedean spirals are similar to those of regular filaments. In essence, if we consider that a filament hugging itself to advance at a roughly constant rate and that it is approximately following a circle of ever increasing radius *r*, then *r* will increase with the square root of time.

Filaments consist of thin tubes where unreacted inner fluid flows, encased by precipitate walls on the sides. A thin membrane exists at the tip of the active advancing filament. Previous research on these structures considers the variation of concentration of precipitate product at the tip, *c*, affected by the accumulation due to reaction and the spreading due to outflow, as well as the internal pressure at the tip, *p*, which changes with the variation of volume of fluid in the filament and the deformation of the membrane. Modeling suggests that these parameters oscillate during the evolution of the filament, leading to its characteristic zigzag motion. The model can be derived to its non-dimensional form,^27,28^ presented here:
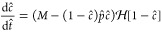
17a
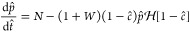
17bwhere  is the Heaviside function.

This system is defined by three dimensionless groups:
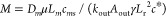
is a nondimensional rate of accumulation of solid;
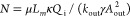
is a nondimensional volumetric injection rate of metal ion;
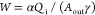
measures the pressure drop along the filament. *ĉ*, *p̂*, and *t̂* are the nondimensionalized variables for the concentration of product, internal pressure and time, obtained from the nondimensionalizing scales *c*_s_ = c*, *p*_s_ = *γA*_out_/κ, and *t*_s_ = *μL*_m_/(*γA*_out_*k*_out_). *D*_m_ is the diffusion coefficient, *L*_m_ the membrane thickness, *L*_r_ the length scale of reaction, *k*_out_ the membrane permeability, μ the fluid viscosity, *A*_out_ the cross-sectional area of the filament, γ the membrane deformation, κ the curvature of the membrane [defined as κ = 1/(*H*/2)], α the pressure drop along the filament, c* the critical product concentration, *c*_ms_ the inner fluid concentration, and *Q*_i_ the flow rate inside the filament. For filaments grown with cobalt chloride and sodium silicate, the values of *L*_m_, *k*_out_, and *L*_r_^2^c* have been estimated as 4.8 × 10^–6^ m, 1.6 × 10^–10^ m^2^, and 3.4 × 10^–8^ mol m^–1^, respectively.^27^ The tortuous motion of the filament tip, periodically changing direction, is associated with a frequency of oscillation, *f*. This variable can be derived from the model as
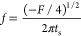
18where
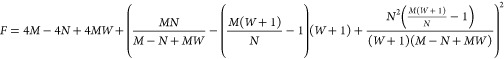
19

For regular filaments, this frequency of oscillation can be estimated experimentally from the speed of the filament tip, *u*_t_, and the typical distance between turns, δ, as *f* = *u*_t_/δ. This allows the comparison of the theory with experiments. In the spirals, however, no turns exist because these hug the candy floss growth and thus follow the direction of its outline. This hugging motion may be due to the local concentration of ions: as the candy floss spreads, it consumes the silicate ions in the area in front of it; as the spiral loops around and arrives at that region, its inner side will face a relatively depleted area of ions, compared to the outer side. This asymmetry makes it more likely for a deformation to occur on the inner side of the filament because the precipitate membrane is bound to be weaker on that side. As a result, the segments will remain “tied” to the candy floss structure because they are unlikely to turn toward the outside bulk silicate solution. In spite of this, the dynamics of the spiral segments should not be fundamentally different from that of a regular filament: a thin precipitate membrane will exist at the tip, and the parameters *c* and *p* will continuously oscillate. Because the path of the spiral is fixed, these dynamics should then just affect their width in a periodic manner: the precipitation reaction concentrates the product in the membrane, narrowing the segment; outflow spreads the precipitate and expands the width of the spiral channel. Indeed, the spiral segments do not have a constant width, with slight bumps on their outline, as shown in Figure 8a. We assume that these are due to the oscillatory dynamics of chemical garden filaments and that the average gap between these bumps is analogous to the typical distance between turns in a filament, δ.

In a similar fashion to the study of the traditional filaments, experimental measurements of the frequency of oscillation *f* can then be compared with theoretical predictions calculated with eq 18. The main challenge in this analysis lies with the fact that the exact flow rate inside the spiral segments is unknown (it is a fraction of the flow rate pumped into the cell). The low flow rates involved are also at the limit of applicability of the model.^27^ Furthermore, candy floss generally spreads as an irregular circular shape; because the spiral follows the outline of the candy floss structure, some turns and bumps may be not be due to the oscillatory dynamics. Nevertheless, an attempt is made to estimate the characteristics of the Archimedean spirals in order to investigate the possibility the model may be applicable. Assuming a flow rate inside the spiral segments in the range of 4.2–5.8 μL s^–1^ (0.25–0.35 mL min^–1^) for the higher flow rate experiments, and a segment width of 0.9 mm, eq 18 yields frequencies of oscillation ranging from 8.7 to 4.6 Hz. These values of *f* are just slightly higher than those of filaments generated with the same chemical system, which is consistent with the similar speed of the active tip in both cases and the fact that the bumps in the spirals are more closely spaced than the turns in the filaments. This supports the possibility that the spirals are indeed filaments self-hugging the candy floss area.

Confirmation of this hypothesis will require more accurate measurements and data from the spirals. A high speed camera may be used to record the development of the structure; modeling predicts the bumps along their outline are due to a periodical variation of the width of the moving tip of the spiral, independent from irregularities in the edge of the expanding candy floss. Additionally, direct and accurate measurements of the dimensions and properties of the precipitate membranes could allow for further information on the similarities or differences between spirals and regular filaments, as well as confined chemical gardens as a whole.

## 5 Results and Discussion

**Figure 10:**
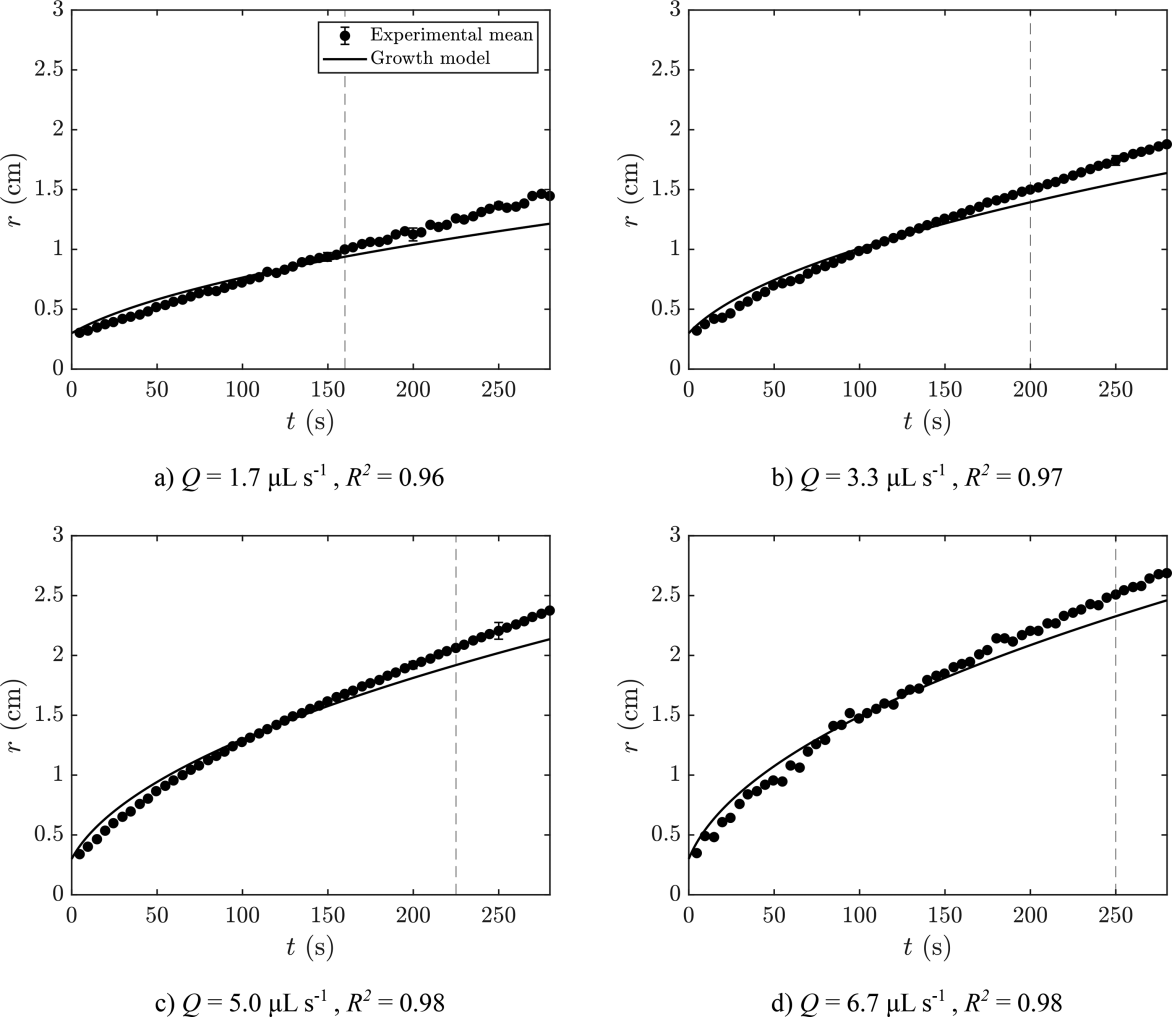
Plots of experimental radius as a function of time compared with a theoretical model for a compact system and conservation of volume for *Q* = 1.7–6.7 μL s^–1^ (*Q* = 0.1–0.4 mL min^–1^). An effective density ρ_eff_ = 1.19 × 10^3^ kg m^–3^ was used for all. For each experiment, *r* = 0.3 cm at *t* = 0 s, which corresponds to the radius of the nozzle.

The precipitate growth model can be compared with the experimental results obtained with the image analysis method described in section 2 and the literature.^26,27^ The two initially unknown parameters are the effective density of the precipitate, ρ_eff_, and the membrane’s permeability, *k*. The effective density was assumed constant across the entire area of solid and was estimated experimentally as presented in section 3.3. This leaves the permeability as the fitting parameter of the model to the experimental data. The experimental results for the growth of precipitate are shown in Figure 10 for all injected flow rates tested. The data are shown together with the respective model prediction. The data are presented only for the Archimedean spiral and candy floss regimes and show a good agreement between experiments and model. During the lichen/worms regimes, the model underestimates the growth of precipitate likely because of the formation of unreacted pockets of sodium silicate within the structure, which are unaccounted for by the image analysis method.

Despite the good performance of the model, it must be noted that in all cases the model initially overestimates the growth of solid and then underestimates it. This suggests that ρ_eff_ and *k* are not constant as the chemical garden evolves. This trend implies that the effective density decreases with time, the permeability increases, or both at the same time. This possibility is consistent with the appearance of inclusions of sodium silicate in later stages of growth, as shown in Figure 8b. These are difficult to measure accurately and not taken into account by the image analysis method, leading to the area of precipitate being overestimated.

The model also considers the simplest assumption that the product occupies the whole gap of the cell, essentially as a cylinder of radius *r* and height *H*. However, given the larger density of the precipitate compared to the solutions, it may sink to the bottom of the cell, leading to a decrease in the actual value of *H* as the structure expands. This effect is likely to be more important in the regions with visible silicate entrapments. The model may be expanded accordingly with accurate measurements of this sinking effect on the effective value of *H*.

The transition from candy floss to lichen/moss then corresponds to the change from a compact system, which naturally grows with the square root of time, to a noncompact system with elongated fingers and entrapments, which approaches linear growth. The model considers only the evolution of a compact system, hence the discrepancy at longer times. Further experiments to determine the actual density of chemical garden membranes and their permeability can clarify this question.

The values of the fitting parameter of the model, the permeability *k*, are presented in Table 1 as a function of flow rate. Permeability increases in a seemingly linear way with injection flow rate into the cell; a linear regression of the data yields the relationship *k* = 15.9*Q* + 0.4 (*R*^2^ = 0.99), where *k* has units of 10^–16^ m^2^ and *Q* has units of mL min^–1^.

**Table 1:** Chemical Garden Permeability (*k*) for Each Flow Rate Studied, Obtained from Fitting the Model to the Experimental Results

*Q* (mL min^–1^)	*k* (×10^–16^ m^2^)
0.1	1.82 ± 0.07
0.2	3.87 ± 0.15
0.3	5.05 ± 0.27
0.4	6.73 ± 0.35

As the flow rate is reduced, the system is expected to become more similar to chemical gardens grown from pellets. In these experiments, there is no injection and the precipitate growth is driven by osmotic pressure only. Permeabilities of such membranes have been reported to lie in the range *k* = (4.6–27) × 10^–19^ m^2^.^40^ The upper range of these values is just 1 order of magnitude below the expected permeability for zero flow rate, which supports the validity of these results; it is likely the permeability will not always vary linearly with flow rate. At higher flow rates filaments are formed, which exhibit a much higher local velocity. In the filament regime, the combination of reaction and flow lead to a continuous “opening and closing” of the membrane, maintaining it at a low width and much higher permeability. Indeed, in the filament regime, permeability of the membrane has been estimated to be of the order of 10^–10^ m^2^, several orders of magnitude higher than the permeabilities estimated here.

## 6 Conclusions

The growth of confined chemical gardens created with low injection flow rates was investigated and modeled mathematically. In accordance with the literature, new patterns are formed as the local velocity decreases, starting with Archimedean spirals, then candy floss, and finally lichen/worms. The candy floss and Archimedean spiral patterns are novel regimes identified in this work, with the latter possibly the result of a filament traveling along the edge of the precipitate with a self-hugging motion. Further study of this regime may involve obtaining more accurate data of the speed of the spirals and the frequency of formation of the bumps along their outline.

The effective density of the solid system was estimated experimentally to be ρ_eff_ = (1.19 ± 0.07) × 10^3^ kg m^–3^, and the fitting of a model to the experimental results for growth yielded membrane permeabilities in the range *k* = (1.8–6.7) × 10^–16^ m^2^, found to increase linearly with increasing injection flow rate. Despite good agreement between theory and experiment, discrepancies suggest that ρ_eff_ and *k* vary during the expansion of the solid, thus requiring further study to be accurately measured.

Spirals have previously been noted in the growth of confined chemical gardens.^18^ But those are logarithmic spirals and form by a completely different mechanism of the curvature of a growing membrane under pressure in a part of the parameter space far from that which we have considered in this work. Archimedean spirals, or the near identical curve, the involute of the circle, that tends very fast to an Archimedean spiral,^32−34^ appear in natural growth processes like the formation of bees combs^41^ and nacre (mother of pearl).^42,43^ In particular, they appear in the growth of many crystals through the Burton–Cabrera–Frank mechanism^44^ of growth at a screw dislocation in a crystal lattice. Likewise, they appear as spiral waves in chemical oscillators like the Belousov–Zhabotinsky reaction.^45,46^ However, the growth mechanism we have identified here, at the mesoscale, although it also leads to an Archimedean spiral, differs from that one. The crystal growth mechanism produces spiral terraces that may have equal width, from much smaller growth units—atoms or molecules—that come together in a fashion that may be described as a type of excitable system.^47^ Our mechanism here, on the other hand, is that of coiling rope, rolls of paper, and so on, in which the Archimedean spiral forms through the advance of a constant width filament, rope, or sheet that hugs itself.
